# A Double Diagnosis Behind Chronic Ulcers: Livedoid Vasculopathy With Acroangiodermatitis in an Elderly Woman

**DOI:** 10.7759/cureus.106073

**Published:** 2026-03-29

**Authors:** Binod Bekoju, Prakash Velmurugan, Sunandan Bhattacharya, Anya Rehman-Wood, Anushka Bajgamage

**Affiliations:** 1 General Practice, Medway NHS Foundation Trust, Gillingham, GBR; 2 Internal Medicine, Medway NHS Foundation Trust, Gillingham, GBR; 3 General Internal Medicine, St George's Hospital, Gillingham, GBR; 4 Geriatrics, Medway NHS Foundation Trust, Gillingham, GBR

**Keywords:** acroangiodermatitis, chronic ulcer, chronic venous leg ulcers, dapsone, fibrinoid necrosis, livedoid vasculopathy, mali type acronagiodermatitis, purpuric patch and plaque, thrombotic vasculopathy, vascular ulcer

## Abstract

Chronic leg ulcers are a significant source of morbidity in elderly patients, particularly those with multiple comorbidities. The most common etiologies are vascular, including chronic venous insufficiency, peripheral arterial disease, and diabetic neuropathy. However, ulcers that are non-healing and refractory to standard treatment may reflect rarer underlying pathologies, warranting comprehensive reassessment.

We report the case of an 87-year-old woman presenting with recurrent, painful, bilateral lower-limb ulcers, persisting for 18 months on the right leg and 10 months on the left. Her medical history was notable for chronic kidney disease, heart failure, and a previous myocardial infarction managed with dual coronary artery stenting. The ulcers had been treated in the community with dressings and multiple courses of oral antibiotics, with recurrent hospital admissions for secondary infection.

She re-presented to the emergency department with worsening ulceration, rigours, raised inflammatory markers, and clinical features of infection. Initial management included intravenous antibiotics, analgesia, specialist wound care, and tissue viability input. Vascular surgical assessment confirmed adequate arterial supply, and there was no clinical evidence of peripheral neuropathy. Despite optimal conventional management, wound healing remained poor, prompting consideration of alternative diagnoses, including pyoderma gangrenosum, vasculitic ulceration, and cutaneous malignancy. Extensive autoimmune and vasculitis screening was negative.

Punch biopsy demonstrated a vasculopathic pattern with increased vascular proliferation, fibrinoid necrosis of small vessels, diffuse neutrophilic infiltrates, and fibrin deposition extending into the deeper dermis. These findings were consistent with coexisting livedoid vasculopathy (LV) and acroangiodermatitis (AAD) secondary to chronic venous stasis, excluding other differentials. This combination is rare, particularly in advanced age. Treatment included topical clobetasol 0.05% cream to peri-wound skin, topical potassium permanganate 0.01% to infected areas, oral dapsone 50 mg twice daily for 3 months, and clopidogrel 75 mg daily, alongside compression therapy and multidisciplinary wound care. Marked clinical improvement was observed within two weeks.

This case highlights the diagnostic complexity of chronic leg ulcers, the importance of maintaining a broad differential diagnosis, and the pivotal role of skin biopsy and multidisciplinary collaboration in guiding effective, individualised management.

## Introduction

We present an elderly female who presented with recurrent non-healing ulcers of the lower extremities that were not responding to conventional treatment. On evaluation, she was found to have coexisting acroangiodermatitis (AAD) with livedoid vasculopathy (LV), which is a very rare occurrence.

Acroangiodermatitis

AAD is a stasis dermatitis that simulates Kaposi's disease [1]. It is a benign disease, unlike Kaposi sarcoma [2]. There are two main variants of AAD: 1. Mali-type AAD associated with chronic venous stasis, and 2. Stewart-Bluefarb syndrome with arteriovenous malformation [3].

The mean age of incidence of AAD in patients with chronic venous insufficiency (CVI) is 53 years [4], but the age of onset can be as early as 14 years of age in Stewart-Bluefarb syndrome [5]. The exact prevalence is not known. It is most common in the lower extremities [6] and is usually bilateral when it is associated with CVI [4]. The condition is more common in males; in a review of 34 reported cases, 22 were males as compared to only 12 females [4].

Clinically, patients present with multiple erythematous-violaceous and purpuric patches and plaques [7]. A lesional skin biopsy shows a hyperkeratotic stratum corneum, dilated capillaries with plump endothelial cells, extravasated red blood cells, and hemosiderin deposits surrounded by hyperplastic granulation tissue with a perivascular mononuclear infiltrate in the upper dermis, with no vascular slits. Perls' stain demonstrates hemosiderin deposits [8].

Treatment includes systemic therapy with dapsone, erythromycin, or doxycycline, along with elastic bandages and topical corticosteroid ointments [9].

Livedoid vasculopathy

LV is a thrombotic vasculopathy of the dermis [10]. It is characterised by chronic, recurrent ulcers and thromboembolic disease with occlusions of dermal vessels [11]. LV is a non-inflammatory thrombotic condition that may present as a primary (idiopathic) or secondary subtype associated with abnormal coagulation factors [12]. Lesions commonly occur on the lower extremities, typically below the knees [13], and are usually bilateral [14].

The condition is very rare, and the estimated prevalence is 1 in 100,000 [10]. It predominantly affects females, with a mean age of presentation of 31 years; however, the age of onset can range from 11 to 85 years [15].

Clinically, it may present with the triad of livedo racemosa, ulcerations on the distal legs, and atrophie blanche, although the triad is not necessary for diagnosis [16].

Histopathological features include focal thrombosis of distal dermal vessels, most commonly affecting capillaries. The lesions are usually superficial and rarely involve subcutaneous fat. Unlike vasculitis, LV shows only minimal lymphocytic infiltration. Studies have demonstrated complement and immunoglobulin deposition (C3>IgM>IgG>IgA) in vessel walls; this finding is not specific for LV [10].

Treatment includes general measures such as wound care, smoking cessation, and compression therapy. Pharmacological therapy may include antiplatelet agents such as aspirin, dipyridamole, and pentoxifylline [17]. Recently, anticoagulants, particularly rivaroxaban, have been shown to be effective in the treatment of LV [18].

There is an increased incidence of LV in patients with venous stasis [1]. Additionally, AAD may occur in the setting of chronic venous insufficiency [17], which itself can develop as a consequence of chronic venous stasis. This association suggests a potential pathophysiological link between these two conditions.

## Case presentation

An 87-year-old woman, who lived with her spouse and was usually mobile with the aid of a Zimmer frame, presented with a chronic history of painful leg ulcers. The ulcer on the right leg had been present for 18 months, while the ulcer on the left leg had persisted for 10 months. The patient has a background medical history of chronic kidney disease, heart failure, and a previous myocardial infarction treated with double coronary stenting. She was initially managed in the community by her general practitioner and received three courses of oral antibiotics in addition to community-based wound care. Over the preceding year, she had multiple hospital admissions due to recurrent infections of the leg ulcers.

The patient subsequently re-presented to the emergency department with worsening bilateral painful ulcers over both shins, complicated by secondary infection. This was evidenced by elevated inflammatory markers and associated rigours. Wounds were obtained from the infected ulcers, and intravenous antibiotics were commenced. She was then transferred to a specialised elderly medicine service for ongoing management.

**Figure 1:**
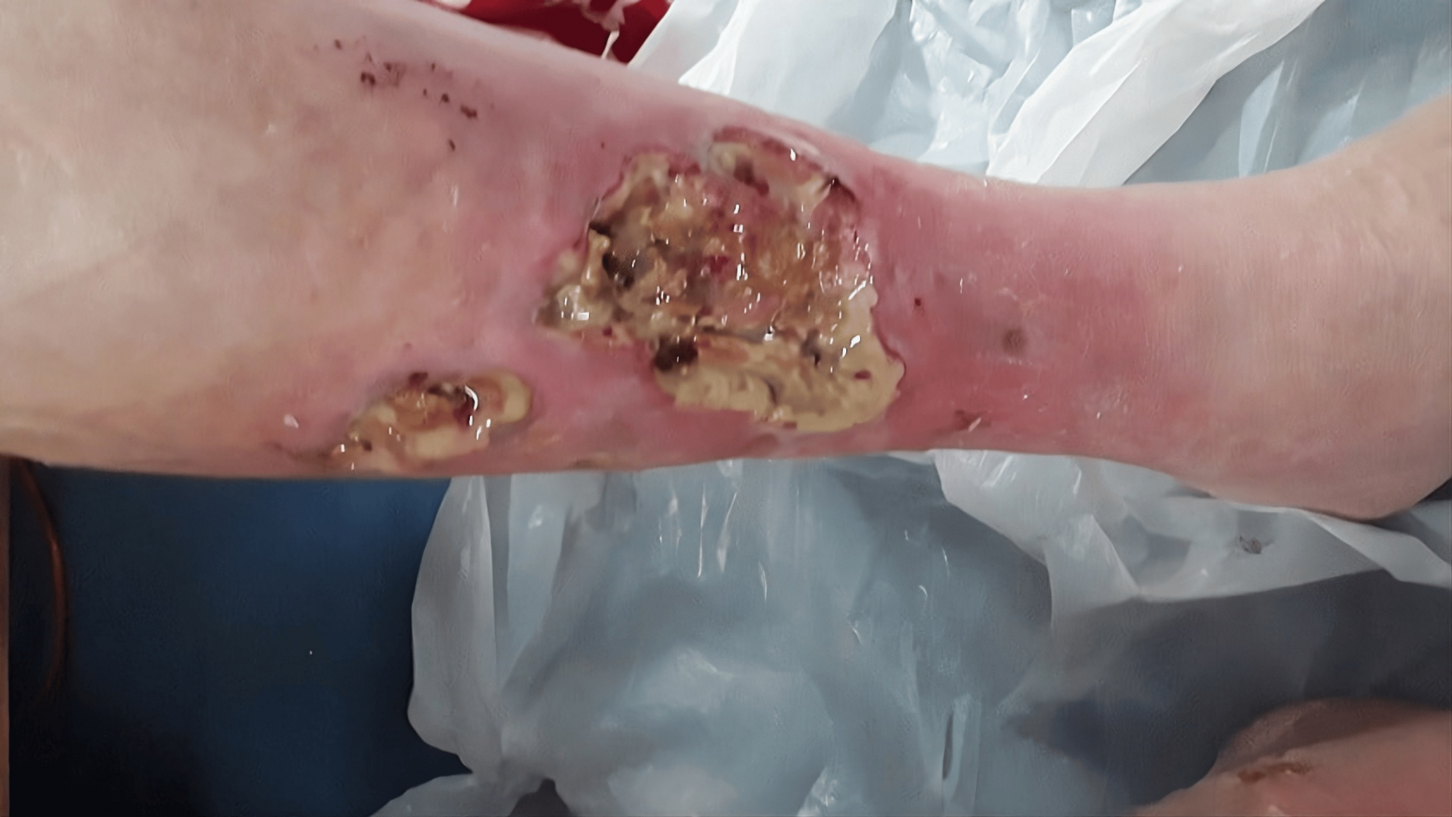
Left leg ulcer Showing an unhealthy, chronic, deep, leg ulcer at the anteriomedial aspect of the left leg with exudate, slough and necrotic debris in the base and surrounding inflamed skin

On examination, the left leg dressing was stained with blood and was foul-smelling. There was a 4x3 cm ulcer on the medial aspect of the left lower leg and a 5x3 cm ulcer on the anterior aspect, with a few smaller ulcers present (Figure 1).

**Figure 2:**
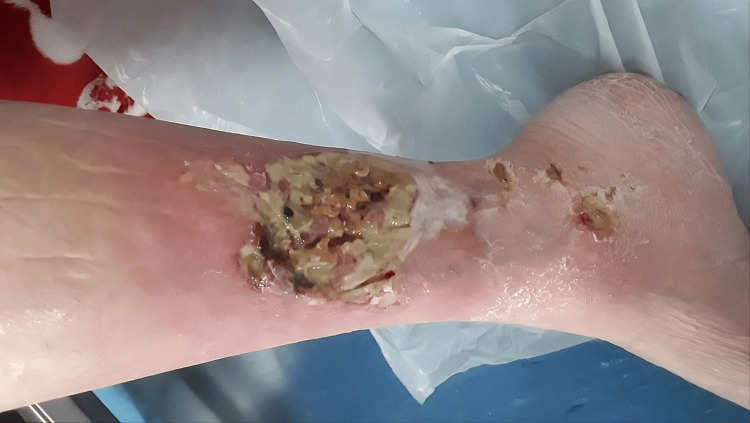
Right leg ulcer Large, deep chronic leg ulcer at the lower third of the leg above the medial malleolus covered with purulent discharge, slough and necrotic debris

Multiple small ulcers, the largest ulcer being 3x2 cm, with greenish crusting around the margins and a foul smell, were seen on the right leg. The ulcer was painful, purplish, and undermined with some discharge and slough (Figure 2).

She was initially managed with intravenous antibiotics and pain killers, and regular wound care was given, resulting in an improvement in CRP (Table 1). Wound culture revealed Streptococcus dysgalactiae, Bacteroides fragilis, enterococci and mixed coliform, sensitive to metronidazole, penicillin, and cefalexin. Antibiotics were adjusted according to the sensitivity result and the microbiologist's advice. She was referred to the tissue viability team, and local ulcer management protocols were implemented. During her hospital stay, she was reviewed by the vascular surgery team, who confirmed adequate arterial blood supply and excluded arterial insufficiency. Clinical examination revealed no evidence of peripheral neuropathy; therefore, no further neurological investigations, such as nerve conduction studies, were undertaken.

**Table 1:** Trend of CRP values during the hospital stay

Date	25/07/25	28/07/25	30/07/25	01/08/25	02/08/25	04/08/25	05/08/25	07/08/25	09/08/25	10/08/25	12/08/25	
CRP Value	67.2	146.2	151.4	201.4	211.5	173.9	175.6	164.6	63.7	26.2	14	

However, wound healing remained unsatisfactory, prompting the medical team to consider alternative differential diagnoses. The differentials included vasculitis lesions, pyoderma gangrenosum, and cutaneous malignancy, given the atypical nature of the ulcers and their poor response to conventional treatment. Accordingly, further investigations were undertaken to evaluate these potential diagnoses.

Autoimmune screening, vasculitis screening, and systemic infection, such as HIV, screening, were performed to rule out autoimmune conditions and vasculitis causes. The medium antinuclear antibody (ANA) positive status was discussed with the rheumatologist, who, after careful review of the clinical findings and other blood investigations, suggested a biopsy of the skin lesions to confirm the exact pathology, since the investigations for thrombophilia could not be carried out due to logistical reasons. Immunohistochemistry done from the groin lymph node biopsy revealed a benign non-specific reactive lymphadenopathy (Table 2 and Table 3).

**Table 2:** Microbiological tests to rule out a systemic infectious cause for the chronic non-healing ulcer

Investigation	Results
Blood culture	No growth
Urine culture	No growth
HIV	Negative
Hepatitis B	Negative
Hepatitis C	Negative

**Table 3:** Autoimmune serology showing the absence of relevant autoimmune antibodies for the patient presentation

Gastric Parietal Cell Ab	Negative
Anti-Smooth Muscle	Negative
Mitochondrial Ab	Negative
LKM antibody titre	Negative
Ant Nuclear Ab	Medium positive (1:160-320)
ANA Pattern	Homogeneous and speckled
dsDNA Ab (Phadia)	Negative
ENA Ab Screen	Negative

The non-healing nature of the ulcers prompted referral to the dermatology team, who arranged a skin biopsy to exclude the differential diagnoses and establish a definitive diagnosis.

Biopsy findings

Punch biopsies demonstrated a vasculopathic pattern, characterised by increased vascular proliferation and extensive fibrinoid necrosis of the walls of multiple small blood vessels in the upper and mid-dermis. This was accompanied by a diffuse neutrophilic infiltrate and fibrin deposition, with involvement extending into the deeper portions of the biopsy specimen. These histopathological features were consistent with elements of LV associated with AAD secondary to chronic venous stasis and chronic venous ulceration. The findings effectively excluded pyoderma gangrenosum. Given the rarity of this presentation, the results were discussed with the dermatology team, and management was guided according to evidence-based practice.

The patient was treated with topical clobetasol 0.05% cream twice daily around the wound, topical potassium permanganate 0.01% twice daily over the infected wound, antiplatelet clopidogrel 75 mg once daily, and dapsone 50 mg twice daily for three months, along with supportive care. There was significant improvement in wound healing within two weeks, and the patient was discharged and referred to the dermatology clinic for follow-up care in the community and blood monitoring for agranulocytosis, as she had been started on dapsone.

## Discussion

Chronic leg ulcers are a common cause of morbidity among elderly patients, with vascular aetiologies, including chronic venous insufficiency, peripheral arterial disease, and diabetic neuropathy, being the most frequent contributors. In the present case, the patient’s chronic non-healing ulcers were initially considered recurrent infected venous ulcers and were managed with dressings and antibiotics. The lack of clinical improvement prompted a multidisciplinary review involving the tissue viability team, vascular surgery, and a specialist dermatology opinion. Initial differential diagnoses included infected leg ulcers, pyoderma gangrenosum, and vasculitic ulceration. The absence of systemic features, in combination with negative autoimmune and vasculitis screening, excluded systemic autoimmune disease or vasculitis, while adequate peripheral perfusion ruled out most vascular differentials. Definitive diagnosis was achieved through punch biopsy histopathology, which demonstrated features consistent with coexisting LV and AAD, a rare occurrence in this age group.

Management of LV primarily focuses on improving microcirculation, reducing thrombosis, and promoting wound care. Thus, management involves anticoagulant or antiplatelet therapy, while dapsone is effective for AAD. The patient was maintained on antiplatelet therapy for LV, as anticoagulation could not be initiated due to a history of bleeding ulcers. She was also started on dapsone and topical steroids for AAD along with meticulous wound care, infection control, compression therapy, and pain management.

Studies by Chhabra et al. demonstrated that AAD occurs more frequently in males [5]. In contrast, a 10-year retrospective analysis conducted by Gao et al. reported that LV predominantly affects younger females [15]. In the present case, our patient was diagnosed with both conditions; however, the age and sex did not align with the demographic patterns previously described in the literature. Such an atypical presentation may lead clinicians to overlook one of the diagnoses or fail to consider the possibility of coexistence.

This case report emphasises the critical role of histopathological examination in the evaluation of recurrent, chronic, non-healing ulcers. Accurate diagnosis is essential, as the management strategies for AAD and LV differ significantly. Early biopsy and careful clinicopathological correlation are therefore crucial to ensure appropriate treatment and improved patient outcomes.

Limitations of the study

This study has several limitations. First, long-term follow-up was not feasible due to the patient’s frailty and multiple comorbidities, which limited the ability to assess sustained outcomes. Second, a thrombophilia workup was not performed because of logistical constraints. Finally, the study lacked objective, standardised metrics for wound healing assessment, which may affect the precision and reproducibility of the reported outcomes.

## Conclusions

This case highlights the diagnostic complexity of chronic leg ulcers in elderly patients with multiple comorbidities. What was initially presumed to be a straightforward infected venous ulcer was ultimately shown, through histopathological evaluation, to represent coexisting livedoid vasculopathy and acroangiodermatitis secondary to chronic venous stasis, necessitating specific management strategies. The case underscores the importance of maintaining a broad differential diagnosis and of performing a biopsy in non-healing ulcers that fail to respond to conventional therapy. A multidisciplinary approach, involving dermatology, vascular surgery, and tissue viability teams, was crucial for establishing an accurate diagnosis and implementing tailored, evidence-based management.
